# Activation of the Extracytoplasmic Function σ Factor σ^P^ by β-Lactams in Bacillus thuringiensis Requires the Site-2 Protease RasP

**DOI:** 10.1128/mSphere.00511-19

**Published:** 2019-08-07

**Authors:** Theresa D. Ho, Kelsie M. Nauta, Ute Müh, Craig D. Ellermeier

**Affiliations:** a Department of Microbiology and Immunology, Carver College of Medicine, University of Iowa, Iowa City, Iowa, USA; b Graduate Program in Genetics, University of Iowa, Iowa City, Iowa, USA; University of Kentucky

**Keywords:** cell envelope, extracellular signaling, gene expression, sigma factors, signal transduction, stress response

## Abstract

Bacteria can utilize alternative σ factors to regulate sets of genes in response to changes in the environment. The largest and most diverse group of alternative σ factors are the extracytoplasmic function (ECF) σ factors. σ^P^ is an ECF σ factor found in Bacillus anthracis, Bacillus cereus, and Bacillus thuringiensis. Previous work showed that σ^P^ is induced by ampicillin, a β-lactam antibiotic, and required for resistance to ampicillin. However, it was not known how activation of σ^P^ is controlled or what other antibiotics may activate σ^P^. Here, we report that activation of σ^P^ is specific to a subset of β-lactams and that σ^P^ is required for resistance to these β-lactams. We demonstrate that activation of σ^P^ is controlled by the proteolytic destruction of the anti-σ factor RsiP and that degradation of RsiP requires multiple proteases. Upon exposure to β-lactams, the extracellular domain of RsiP is cleaved by an unknown protease, which we predict cleaves at site-1. Following cleavage by the unknown protease, the N terminus of RsiP is further degraded by the site-2 intramembrane protease RasP. Our data indicate that RasP cleavage of RsiP is not the rate-limiting step in σ^P^ activation. This proteolytic cascade leads to activation of σ^P^, which induces resistance to β-lactams likely via increased expression of β-lactamases.

**IMPORTANCE** The discovery of antibiotics to treat bacterial infections has had a dramatic and positive impact on human health. However, shortly after the introduction of a new antibiotic, bacteria often develop resistance. The bacterial cell envelope is essential for cell viability and is the target of many of the most commonly used antibiotics, including β-lactam antibiotics. Resistance to β-lactams is often dependent upon β-lactamases. In B. cereus, B. thuringiensis, and some B. anthracis strains, the expression of some β-lactamases is inducible. This inducible β-lactamase expression is controlled by activation of an alternative σ factor called σ^P^. Here, we show that β-lactam antibiotics induce σ^P^ activation by degradation of the anti-σ factor RsiP.

## Abstract (precis)

The discovery of antibiotics to treat bacterial infections has had a dramatic and positive impact on human health. However, shortly after the introduction of a new antibiotic, bacteria often develop resistance. The bacterial cell envelope is essential for cell viability and is the target of many of the most commonly used antibiotics, including β-lactam antibiotics. Resistance to β-lactams is often dependent upon β-lactamases. In B. cereus, B. thuringiensis, and some B. anthracis strains, the expression of some β-lactamases is inducible. This inducible β-lactamase expression is controlled by activation of an alternative σ factor called σ^P^. Here, we show that β-lactam antibiotics induce σ^P^ activation by degradation of the anti-σ factor RsiP.

## INTRODUCTION

The bacterial cell envelope is essential for cell viability and is the target of many of the most commonly used antibiotics, including β-lactams like penicillins, penems, and cephalosporins. These are broad-spectrum antibiotics that target peptidoglycan (PG) biosynthesis by inhibiting the transpeptidase activity of penicillin-binding proteins (PBPs). This results in decreased and/or altered cross-linking of peptidoglycan, which leads to cell envelope damage and subsequent cell lysis and death (1, 2).

Members of the Bacillus cereus group, including Bacillus thuringiensis and Bacillus cereus and some strains of Bacillus anthracis, are highly resistant to β-lactam antibiotics (3–6). This resistance is due in part to expression of at least two β-lactamases (3, 5). The expression of these β-lactamases is induced by ampicillin and is dependent upon the alternative σ factor σ^P^. σ^P^ belongs to the extracytoplasmic function (ECF) family of alternative σ factors (5).

Bacteria often utilize alternative σ factors to regulate subsets of genes required for survival under specific environmental conditions or for stress responses. ECF σ factors are the largest and most diverse group of alternative σ factors and represent the “third pillar” of bacterial signal transduction (7, 8). ECF σ factors belong to the σ^70^ family, but unlike the “housekeeping” σ factor, σ^70^, ECF σ factors contain only region 2 and region 4.2 of σ^70^, which recognize and bind to the −10 and −35 regions of promoter sequences, respectively (8, 9). In addition, unlike σ^70^, ECF σ factors are generally held inactive by anti-σ factors until bacteria encounter an inducing signal (10, 11). Upon induction, ECF σ factors are released from their cognate anti-σ factors to promote transcription of specific stress response genes.

The ECF σ factors have been subdivided into more than 40 distinct groups, with ECF01 being the best studied (reviewed in references 7, 11, and 12). σ^P^ belongs to the ECF01 family, which includes members like σ^E^ and σ^W^ from Escherichia coli and Bacillus subtilis, respectively. The activities of the ECF01 family are inhibited by their cognate transmembrane anti-σ factors (8, 13). To activate ECF01 σ factors, the anti-σ factors must be destroyed via a proteolytic cascade (14, 15). For example, the E. coli anti-σ factor RseA is degraded in response to outer membrane stress, leading to σ^E^ activation (16, 17). DegS, a serine protease, cleaves the anti-σ factor RseA at site-1 (14, 18, 19). After site-1 cleavage, the conserved site-2 protease, RseP, cleaves RseA within the membrane, leading to increased σ^E^ activity (14, 20, 21). Similarly, the σ^W^ anti-σ factor, RsiW, from B. subtilis is proteolytically degraded by site-1 and site-2 proteases. In the case of RsiW, the site-1 protease is PrsW, a metalloprotease unrelated to DegS. PrsW cleaves RsiW in response to antimicrobial peptides, vancomycin, and pH change (22–24). RsiW is further processed by the conserved site-2 protease RasP, a homolog of RseP (15).

The closely related ECF30 family member σ^V^ from B. subtilis is activated by lysozyme (25–29). Activation of σ^V^ differs from σ^E^ and σ^W^ activation in that σ^V^ is not controlled by a dedicated site-1 protease but instead utilizes signal peptidases (30, 31). Signal peptidases are essential proteases which are required to cleave substrates secreted from the general secretion or twin arginine secretion systems (32–34). The anti-σ factor RsiV binds to lysozyme, which allows signal peptidase to cleave RsiV at site-1 (30, 31). This allows the site-2 protease RasP to cleave RsiV, leading to σ^V^ activation (35).

Previous studies found that σ^P^ is induced by ampicillin (Amp) and that its activity is required for resistance to ampicillin (5). The activity of σ^P^ is inhibited by the transmembrane anti-σ factor RsiP (5, 6). However, whether σ^P^ is activated specifically by ampicillin or more generally by cell wall stress is not known. In B. subtilis, activation of σ^V^ is specific to lysozyme (26, 27), while activation of σ^W^, σ^X^, and σ^M^ is in response to more general cell envelope stress (9, 36, 37). Here, we show that σ^P^ is activated by a specific subset of β-lactams and that this activation occurs via regulated intramembrane proteolysis of the anti-σ factor RsiP.

## RESULTS

### A subset of β-lactams induces σ^P^ activation.

Previously, Koehler and colleagues demonstrated that ampicillin induces expression of the β-lactamase encoded by *bla1* (*hd73_3490*) in a σ^P^-dependent manner in B. thuringiensis and B. cereus (5). Activation of some ECF σ factors is highly specific to an inducing signal, while others are activated by more general cell envelope stress. Thus, we sought to determine the specificity of σ^P^ activation using B. thuringiensis as a model system.

**FIG 1:**
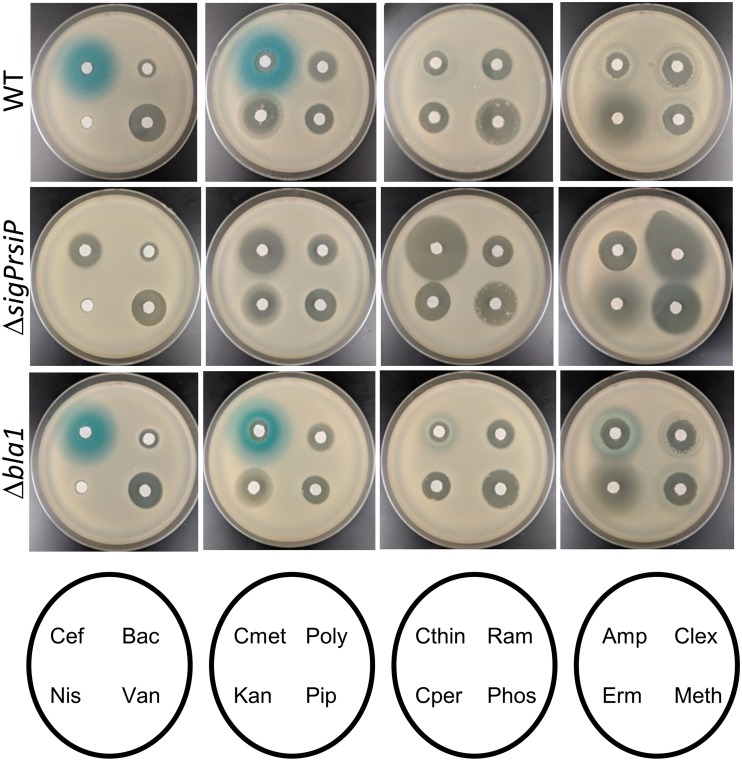
Expression of *sigP* is specifically induced by β-lactams. All the strains contained P*_sigP_*-*lacZ* in either a wild-type (THE2549), a Δ*sigP*-*rsiP* (EBT232), or a Δ*bla1* (EBT215) background. Mid-log cells were washed and diluted 1:100 in molten LB agar containing X-Gal (100 μg/ml) and poured into empty 100-mm petri dishes. Filter disks containing cefoxitin (Cef) (1 μl of 5-mg/ml cefoxitin), bacitracin (Bac) (1 μl of 50-mg/ml bacitracin), nisin (Nis) (3 μl of 100-mg/ml nisin), vancomycin (Van) (1 μl of 10-mg/ml vancomycin), cefmetazole (Cmet) (1 μl of 5-mg/ml cefmetazole), polymyxin B (Poly) (1 μl of 50-mg/ml polymyxin B), kanamycin (Kan) (1 μl of 10-mg/ml kanamycin), piperacillin (Pip) (1 μl of 5-mg/ml piperacillin), cephalothin (Cthin) (1 μl of 50-mg/ml cephalothin), ramoplanin (Ram) (1 μl of 25-mg/ml ramoplanin), cefoperazone (Cper) (1 μl of 50 mg/ml cefoperazone), phosphomycin (Phos) (1 μl of 100-mg/ml phosphomycin), Amp (2 μl of 200-mg/ml ampicillin), cephalexin (Clex) (1 μl of 50-mg/ml cephalexin), Erm (1 μl of 5-mg/ml erythromycin), and methicillin (Meth) (2 μl of 100-mg/ml methicillin) were then placed on the top agar and incubated for 16 h at 30°C.

Like many ECF σ factor systems, σ^P^ is required for its own transcription (5). To monitor σ^P^ activation, we fused the σ^P^ promoter (P*_sigP_*) to the *lacZ* reporter gene and integrated this construct into the genome of B. thuringiensis (THE2549 *thrC*::P*_sigP_*-*lacZ*). We tested several classes of β-lactams and cell wall-targeting antibiotics for their ability to induce expression of P*_sigP_*-*lacZ*. We observed wide zones of P*_sigP_*-*lacZ* induction around cefoxitin and cefmetazole (Fig. 1). We detected fainter zones of induction in the areas around cephalothin and cephalexin (Fig. 1). Very faint zones of induction were present in the cells around ampicillin and methicillin (Fig. 1). Interestingly, we did not observe this induction surrounding the β-lactams cefoperazone and piperacillin or antibiotics that target other steps in cell wall biosynthesis, including ramoplanin, phosphomycin, nisin, bacitracin, and vancomycin (Fig. 1). We also tested compounds that do not target peptidoglycan biosynthesis, including kanamycin, polymyxin B, and erythromycin (Erm), and saw no induction of P*_sigP_-lacZ* (Fig. 1).

**FIG 2:**
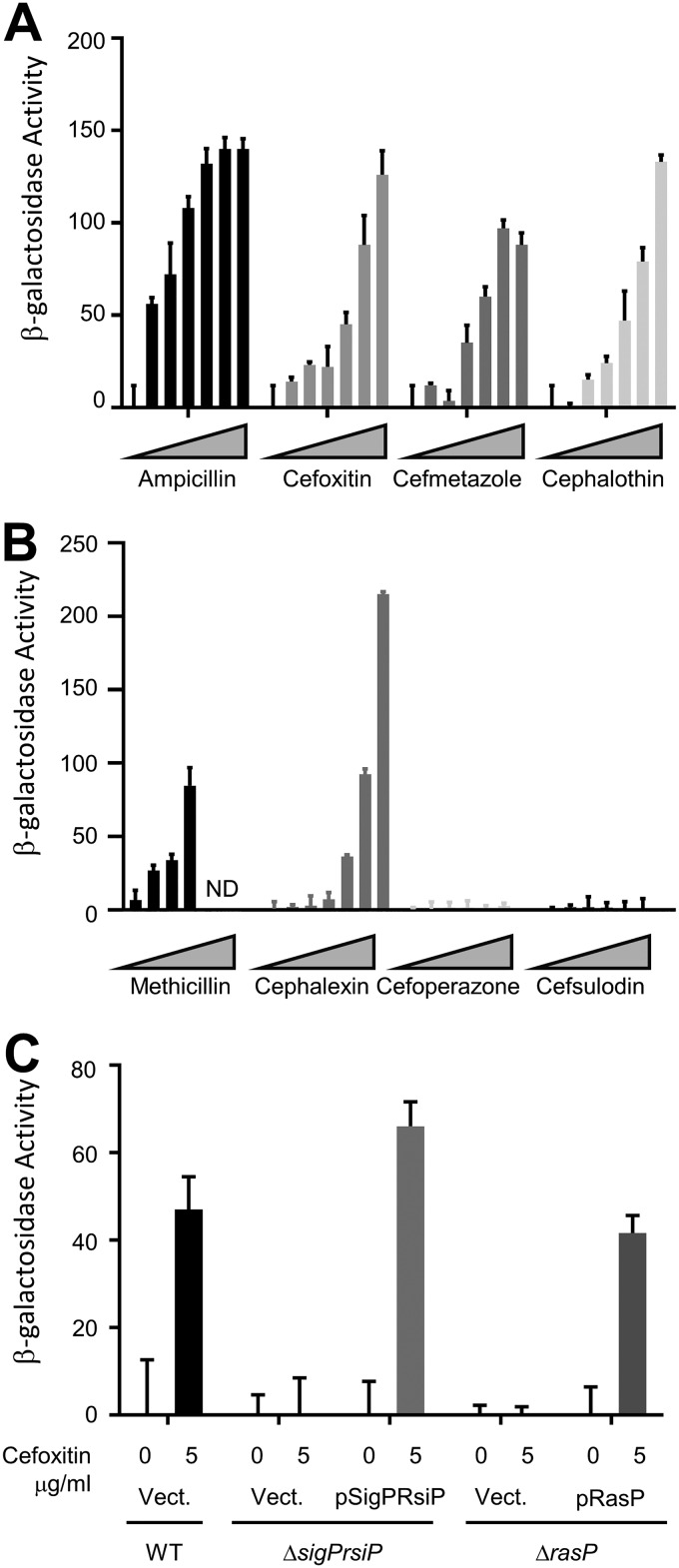
Expression of P*_sigP_-lacZ* is dose dependent and dependent upon σ^P^ and RasP. (A) B. thuringiensis with transcriptional fusion P*_sigP_*-*lacZ* (THE2549) was grown overnight at 30°C, subcultured in LB, and grown to an OD_600_ of ∼0.8 before being incubated with various concentrations of β-lactams (0, 0.0625, 0.125, 0.25 0.5, 1, and 2 μg/ml) for 1 h. Cells were collected and resuspended in Z buffer. (B) B. thuringiensis with transcriptional fusion P*_sigP_*-*lacZ* (THE2549) was grown overnight at 30°C, subcultured in LB, and grown to an OD_600_ of ∼0.8 before being incubated with various concentrations of β-lactams (0, 0.0625, 0.125, 0.25 0.5, 1, and 2 μg/ml) for 1 h. Cells were collected and resuspended in Z buffer. (C) All strains contain P*_sigP_*-*lacZ* and the genotype and plasmid noted: wild type/Vect. (EBT169), *sigP*/Vect. (EBT251), Δ*sigP-rsiP*/pSigPRsiP (EBT238), Δ*rasP*/Vect. (EBT175), and *rasP*/pRasP (EBT176). Strains were grown to mid-log phase and then treated with 5 μg/ml cefoxitin or untreated (0) and incubated for 1 h. β-Galactosidase activity was calculated as described in Materials and Methods. These experiments were done in triplicate, and standard deviations are represented by error bars.

To quantify the levels of β-lactam induction, we tested eight β-lactams for their ability to activate the P*_sigP_*-*lacZ* fusions using a β-galactosidase assay. Mid-log cells were incubated in the presence of various concentrations of ampicillin, cefoxitin, cefmetazole, cephalothin, methicillin, cephalexin, cefoperazone, and cefsulodin for 1 h at 37°C. We observed dose-dependent induction with a subset of these β-lactams (Fig. 2A and B). Interestingly, ampicillin, methicillin, and cephalexin showed low levels of P*_sigP_-lacZ* induction when spotted onto a lawn of cells (Fig. 1) but strongly induced P*_sigP_*-*lacZ* in liquid assays (Fig. 2A and B), a point we will return to later. In contrast, neither cefoperazone nor cefsulodin was able to induce on the plates or in liquid (Fig. 1 and 2B). This confirms our observation that a subset of β-lactams induces σ^P^ activation.

We found that deletion of the *sigP*-*rsiP* genes blocked expression of P*_sigP_-lacZ* in the presence of β-lactams (Fig. 1 and 2C), demonstrating that σ^P^ is required for induction of P*_sigP_-lacZ* in response to β-lactams. When we introduced a low-copy-number plasmid containing P*_sigP_*-*sigP*^+^-*rsiP*^+^ into the Δ*sigP-rsiP* mutant (Δ*sigP-rsiP*/pSigPRsiP), we restored the induction of P*_sigP_*-*lacZ* in response to cefoxitin (Fig. 2C). Taken together, these data suggest that a subset of β-lactam antibiotics activates σ^P^.

### σ^P^ and Bla1 are involved in resistance to some β-lactams.

To determine the impact of σ^P^ on resistance to β-lactams, we measured the MICs of several β-lactams for wild-type and Δ*sigP-rsiP* mutant strains. We found that the wild type was greater than 100-fold more resistant to ampicillin, methicillin, and cephalothin than was the Δ*sigP-rsiP* mutant (Table 1). The wild type was 16- to 50-fold more resistant to cefmetazole, cefoxitin, and cephalexin than the mutant (Table 1). There was little or no difference in resistance to piperacillin, cefoperazone, and cefsulodin, which also failed to activate σ^P^ (Table 1 and Fig. 1). We also demonstrate that complementing the *ΔsigP-rsiP* mutant with a plasmid carrying P*_sigP_*-*sigP^+^*-*rsiP^+^* restored resistance to ampicillin and cefoxitin (Table 2). For reasons that remain unclear, strains containing plasmids, including empty vector, have slight increases in β-lactam resistance. However, this does not impact the observation that the presence of P*_sigP_*-*sigP^+^*-*rsiP^+^* restored resistance to ampicillin and cefoxitin.

**TABLE 1:** *ΔsigP-rsiP* mutant is more sensitive to β-lactams than wild type

Drug	MIC (μg/ml) for strain (mean ± SD):		Fold difference
	WT	Δ*sigP-rsiP* mutant	
Ampicillin	6,000 ± 0	1.67 ± 0.5	3,592
Cefoxitin	200 ± 0	20 ± 0	10
Methicillin	666 ± 115	1 ± 0	666
Piperacillin	5 ± 0	1.25 ± 0	4
Cephalothin	88 ± 25	0.25 ± 0	350
Cephalexin	200 ± 0	4 ± 0	50
Cefmetazole	44 ± 13	2.8 ± 1.1	16
Cefoperazone	5 ± 2	4 ± 0	1.25
Cefsulodin	400 ± 0	400 ± 0	1

**TABLE 2:** RasP is required for resistance to β-lactams^*a*^

Genotype	Vector	MIC (μg/ml) of drug (mean ± SD):		
		Ampicillin	Cefoxitin	Methicillin
WT	Empty	8,000 ± 0	200 ± 0	666.7 ± 115
*ΔsigP-rsiP*	Empty	2 ± 0	20 ± 0	1 ± 0
*ΔsigP-rsiP*	pSigP	6,666 ± 3,011	100 ± 0	ND
*ΔrasP*	Empty	6.7 ± 2.1	20 ± 0	ND
*ΔrasP*	pRasP	6,333 ± 1,966	133 ± 57.7	ND
*Δbla1*	Empty	400 ± 0	200 ± 0	125 ± 50

a Abbreviations: WT, wild type; ND, not determined.

Since σ^P^ was shown to control expression of *hd73_3490* (referred to here as *bla1*), which encodes a β-lactamase, we sought to determine if this gene played a role in resistance to β-lactams. We made a deletion of *bla1* and determined the MIC of ampicillin and cefoxitin for this strain. The *bla1* mutant was 8- to 16-fold more sensitive to ampicillin and ∼5-fold more sensitive to methicillin but no more sensitive to cefoxitin than the wild type (Table 2). This contrasts with the *sigP* mutant, which is greater than 1,000-fold more sensitive to ampicillin, 600-fold more sensitive to methicillin, and ∼25-fold more sensitive to cefoxitin than the wild type (Table 2). This suggests that Bla1 plays a more important role in resistance to ampicillin and methicillin than to cefoxitin. Furthermore, our data suggest that while Bla1 contributes to β-lactam resistance, additional σ^P^-regulated genes must also contribute to β-lactam resistance.

When we tested various β-lactams for induction of P*_sigP_-lacZ* on 5-bromo-4-chloro-3-indolyl-β-d-galactopyranoside (X-Gal) plates, we did not consistently observe a strong zone of induction surrounding ampicillin and methicillin (Fig. 1). We hypothesized that this weak induction zone was due to the wild type efficiently producing β-lactamases which degraded the inducer (ampicillin and methicillin). Thus, we were unable to observe the increased production of β-galactosidase. To test this hypothesis, we determined the effect of a Δ*bla1* mutant on σ^P^ activation. We found that in the Δ*bla1* mutant, ampicillin and methicillin produced more distinct zones of induction (Fig. 1). However, all other induction zones of the Δ*bla1* mutant were similar to the wild type. Thus, in the absence of Bla1, which degrades ampicillin and methicillin, we detected greater induction of P*_sigP_-lacZ* expression. Taken together, these observations suggest that the weak ampicillin induction of P*_sigP_-lacZ* on plates is in part due to the efficient degradation of the inducer by β-lactamases.

### RsiP is degraded in response to cefoxitin in a dose-dependent manner.

The anti-σ factors of other ECF01 family members are degraded, which leads to the activation of their cognate σ factors (7, 14, 15). We sought to determine if β-lactams activate σ^P^ by inducing degradation of RsiP. To investigate this, we constructed a strain with an anhydrotetracycline (ATc)-inducible copy of green fluorescent protein (GFP) fused to the N terminus of RsiP (GFP-RsiP). The inducible promoter allows us to uncouple expression of RsiP from induction of σ^P^. The GFP-RsiP fusion allows us to follow the fate of the cytoplasmic portion of RsiP. Expression of GFP-RsiP complements an *rsiP* null mutation (see Fig. S1 in the supplemental material) and localizes to the membrane (Fig. S2). We then induced the synthesis of GFP-RsiP in exponential-phase cells and monitored its processing before and after treatment with cefoxitin. We chose to utilize cefoxitin for these experiments because cefoxitin induces σ^P^ activation over a wide concentration range and the *ΔsigP-rsiP* mutant strain grows at most of these concentrations (Fig. 2A and Table 1). Cell pellets were then lysed by sonication, and Western blot analyses were performed using anti-RsiP antisera against the extracellular portion of RsiP or anti-GFP antisera, which detect GFP fused to the intracellular portion of RsiP.

10.1128/mSphere.00511-19.1 FIG S1 GFP-RsiP is functional. B. thuringiensis *rsiP*^1–80^ (THE2628) containing tetracycline-inducible *gfp-rsiP* (EBT587) or empty vector (EBT561) was plated on LB–X-Gal without or with ATc (50 ng/ml). Download FIG S1, TIF file, 1.9 MB. Copyright © 2019 Ho et al. 2019 Ho et al. This content is distributed under the terms of the Creative Commons Attribution 4.0 International license.

10.1128/mSphere.00511-19.2 FIG S2 GFP-RsiP localizes to the membrane. B. thuringiensis expressing tetracycline-inducible *gfp-rsiP* (EBT360), tetracycline-inducible *gfp-rsiP*^1–72^ (EBT533), or empty vector (EBT169) was subcultured 1:50 with ATc (100 ng/ml) and grown at 30°C to late log phase. Two microliters was spotted on a 1% agarose pad for immobilization and imaged for GFP localization. Phase-contrast and fluorescence micrographs were recorded on an Olympus BX60 microscope with a 100 UPlanApo objective (numerical aperture, 1.35). For the GFP micrographs, a filter set from Chroma Technology Corp (catalog no. 41017) was used. The GFP filter consists of a 450- to 490-nm excitation filter, a 495-nm dichroic mirror (long pass), and a 500- to 550-nm emission filter. Micrographs were captured with a Hamamatsu Orca Flash 4.0 V2 complementary metal oxide semiconductor (CMOS) camera. Download FIG S2, TIF file, 1.5 MB. Copyright © 2019 Ho et al. 2019 Ho et al. This content is distributed under the terms of the Creative Commons Attribution 4.0 International license.

**FIG 3:**
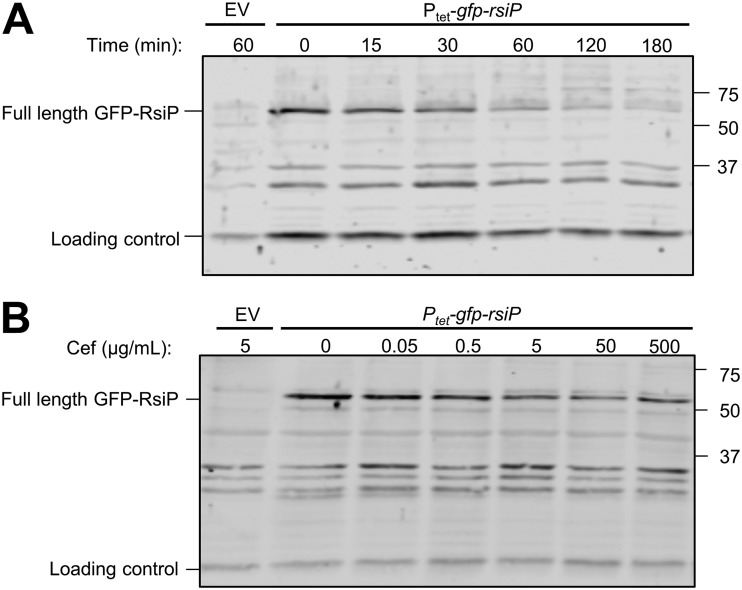
RsiP levels decrease in the presence of cefoxitin. B. thuringiensis expressing tetracycline-inducible *gfp-rsiP* (EBT360) or empty vector (EV; EBT169) was subcultured 1:50 into LB supplemented with ATc (50 ng/ml). At mid-log phase, cells were incubated with 5 μg/ml of cefoxitin for various times (0, 15, 30, 60, 120, or 180 min) (A) or increasing concentrations of cefoxitin (0, 0.05, 0.5, 5, 50, or 500 μg/ml) for 1 h (B). The immunoblot was probed with antisera against RsiP (α-RsiP^76–275^). Streptavidin IR680LT was used to detect HD73_4231 (PycA homolog), which served as a loading control (62, 63). The color blot showing both anti-RsiP and streptavidin on a single gel is shown in Fig. S3. Numbers at right indicate molecular masses in kilodaltons.

When cells producing GFP-RsiP were grown in the absence of cefoxitin, we detected full-length GFP-RsiP at the expected size of ∼60 kDa using anti-RsiP antisera. This band was absent in the empty-vector control (Fig. 3A). When cells were incubated with cefoxitin (5 μg/ml) for various times, we found that the level of full-length GFP-RsiP decreased over time (Fig. 3A and Fig. S3A). We observed loss of GFP-RsiP by 30 min to 1 h after exposure to cefoxitin (Fig. 3A and Fig. S3A). This suggests that GFP-RsiP is likely degraded in the presence of cefoxitin.

10.1128/mSphere.00511-19.3 FIG S3 RsiP levels decrease in the presence of cefoxitin. B. thuringiensis expressing tetracycline-inducible *gfp-rsiP* (EBT360) or empty vector (EBT169) was subcultured 1:50 into LB supplemented with ATc (50 ng/ml). At mid-log phase, cells were incubated with 5 μg/ml of cefoxitin for various times (0, 15, 30, 60, 120, or 180 min) (A) or increasing concentrations of cefoxitin (0, 0.05, 0.5, 5, 50, or 500 μg/ml) for 1 h (B). The immunoblot was probed with antisera against RsiP (α-RsiP^76–275^) followed by goat anti-rabbit IgG IR800CW (green). EV is wild type with pAH9 (EBT169), and GFP is wild type with pAH13 (UM20). Streptavidin IR680LT (red) was used to detect HD73_4231 (PycA homolog), which served as a loading control (62, 63). Download FIG S3, TIF file, 1.5 MB. Copyright © 2019 Ho et al. 2019 Ho et al. This content is distributed under the terms of the Creative Commons Attribution 4.0 International license.

We also tested the effect of cefoxitin concentration on GFP-RsiP levels by incubating cells with a range of cefoxitin concentrations (0 to 500 μg/ml) for 1 h. We found that increasing concentrations of cefoxitin resulted in a greater decrease of full-length GFP-RsiP (Fig. 3B and Fig. S3B). We obtained comparable results when we performed blotting assays for the N-terminal domain using anti-GFP antisera (Fig. S4). These data suggest that activation of σ^P^ occurs via loss of RsiP in a cefoxitin dose-dependent manner.

10.1128/mSphere.00511-19.4 FIG S4 RsiP levels decrease in the presence of cefoxitin. B. thuringiensis expressing tetracycline-inducible *gfp-rsiP* (EBT360), empty vector (EBT169), or GFP alone (UM20) was subcultured 1:50 into LB supplemented with ATc (50 ng/ml). At mid-log phase, cells were incubated with increasing concentrations of cefoxitin (0, 0.05, 0.5, 5, 50, and 500 μg/ml) for 1 h. The immunoblot was probed with antisera against GFP (α-GFP) followed by goat anti-rabbit IgG IR800CW (green). Streptavidin IR680LT (red) was used to detect HD73_4231 (PycA homolog), which served as a loading control (62, 63). The Western blot is shown in black and white (A) or color (B). Download FIG S4, TIF file, 1.9 MB. Copyright © 2019 Ho et al. 2019 Ho et al. This content is distributed under the terms of the Creative Commons Attribution 4.0 International license.

### RasP is necessary for σ^P^ activation.

Both σ^E^ and σ^W^ are activated by regulated intramembrane proteolysis of their cognate anti-σ factors. Proteolysis of these anti-σ factors requires multiple proteases, including the highly conserved site-2 proteases RseP and RasP, respectively (14, 15). We hypothesize that activation of σ^P^ requires multiple proteases, including the conserved site-2 protease RasP to degrade RsiP. To test this, we used BLAST to identify a putative membrane-embedded metalloprotease, HD73_4103, which is 76% similar and 60% identical to B. subtilis RasP and is here referred to as RasP (Fig. S5) (38–43). To determine if RasP was required for σ^P^ activation, we generated a strain containing a deletion of *rasP* and the P*_sigP_*-*lacZ* reporter. In the absence of RasP, we did not detect increased expression of P*_sigP_*-*lacZ* reporter in response to cefoxitin (Fig. 2C). In MIC experiments, we found that, similarly to the Δ*sigP-rsiP* mutant, the Δ*rasP* mutant was more sensitive to ampicillin and cefoxitin (Table 2). We found that both resistance to β-lactams and induction of P*_sigP_-lacZ* could be complemented when a plasmid expressing *rasP^+^* was introduced into the Δ*rasP* mutant (Fig. 2C and Table 2). These data suggest that RasP is required for σ^P^ activation.

10.1128/mSphere.00511-19.5 FIG S5 Amino acid alignment of RasP. An alignment of B. subtilis RasP and B. thuringiensis HD73_4301. The active site is marked by a red box. Download FIG S5, TIF file, 1.3 MB. Copyright © 2019 Ho et al. 2019 Ho et al. This content is distributed under the terms of the Creative Commons Attribution 4.0 International license.

### RasP is required for degradation of RsiP.

**FIG 4:**
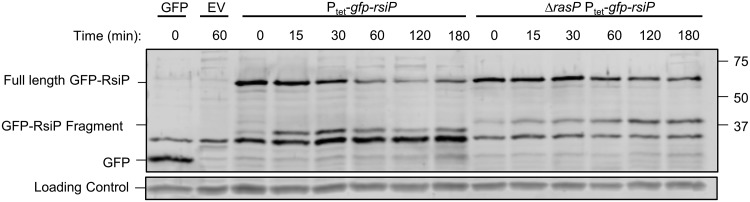
RsiP degradation is dependent upon the site-2 protease RasP. B. thuringiensis wild type (EBT360) or *ΔrasP* (EBT366) containing a tetracycline-inducible copy of *gfp*-*rsiP* was subcultured 1:50 into LB supplemented with ATc (50 ng/ml). At mid-log phase, cultures were incubated with cefoxitin (5 μg/ml) for the time indicated at 37°C. The immunoblot was probed with anti-GFP antisera. EV is wild type with pAH9 (EBT169), and GFP is wild type with pAH13 (UM20). Streptavidin IR680LT was used to detect HD73_4231 (PycA homolog), which served as a loading control (62, 63). The color blot showing both anti-GFP and streptavidin on a single gel is shown in Fig. S6. Numbers at right are molecular masses in kilodaltons.

To determine if RasP is required for degradation of RsiP, we expressed the GFP-RsiP fusion in both the wild type and a *ΔrasP* mutant. We treated cells with 5 μg/ml cefoxitin for various lengths of time from 0 to 180 min (Fig. 4 and Fig. S6). In the wild type, we observed loss of full-length RsiP over time (Fig. 4 and Fig. S6). In contrast, we observed loss of full-length GFP-RsiP and the accumulation of a smaller ∼35-kDa band in the Δ*rasP* mutant (Fig. 4 and Fig. S6). This suggests that RasP is required for complete degradation of RsiP. Since a truncated product accumulates in the *ΔrasP* mutant, RasP is likely required for site-2 cleavage and an unidentified protease is required for cleavage at site-1.

10.1128/mSphere.00511-19.6 FIG S6 RsiP degradation is dependent upon the site-2 protease RasP. B. thuringiensis containing a tetracycline-inducible copy of *gfp*-*rsiP*, wild type (EBT360) or *ΔrasP* (EBT366), was subcultured 1:50 into LB supplemented with ATc (50 ng/ml). At mid-log phase, cultures were incubated for 1 h without (−) or with (+) cefoxitin treatment (5 μg/ml) at 37°C. The immunoblot was probed with antisera against GFP (anti-GFP) followed by goat anti-rabbit IgG IR800CW (green). EV is wild type with pAH9 (EBT169), and GFP is wild type with pAH13 (UM20). Streptavidin IR680LT (red) was used to detect HD73_4231 (PycA homolog), which served as a loading control (62, 63). Download FIG S6, TIF file, 2.7 MB. Copyright © 2019 Ho et al. 2019 Ho et al. This content is distributed under the terms of the Creative Commons Attribution 4.0 International license.

### Mutations in *rsiP* result in constitutive *sigP* expression.

**FIG 5:**
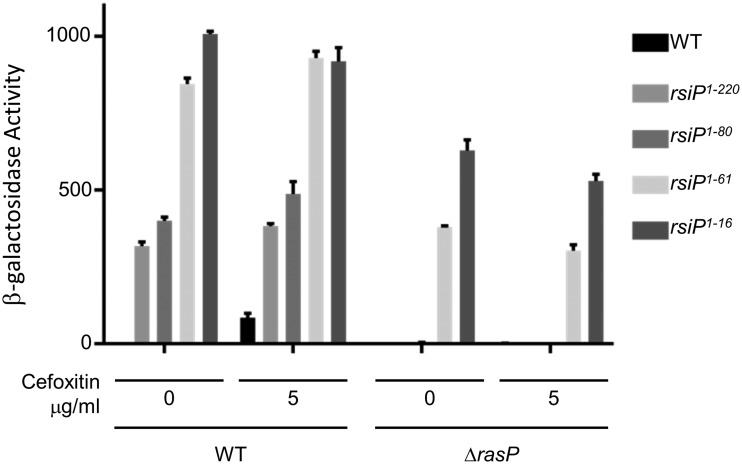
Truncations of RsiP lead to constitutive σ^P^ activation. To determine if RasP was required for ampicillin-inducible P*_sigP_-lacZ* expression, we assayed β-galactosidase activity of B. thuringiensis with transcriptional fusion P*_sigP_*-*lacZ* and different *rsiP* truncation mutants (WT, THE2549; RsiP^1–220^, THE2602; RsiP^1–80^, THE2628; RsiP^1–61^, THE2637; RsiP^1–16^, THE2642) and a Δ*rasP* deletion (WT, EBT140; RsiP^1–220^, EBT116; RsiP^1–80^, EBT148; RsiP^1–61^, EBT133; RsiP^1–16^, THE2605). Cells were grown overnight at 30°C, subcultured in LB, and grown to an OD_600_ of ∼0.8 before being incubated with cefoxitin (5 μg/ml) for 1 h. The experiment was performed in triplicate, and standard deviations are represented by error bars.

To further characterize the σ^P^ signal transduction system, we isolated mutants which resulted in constitutive expression of P*_sigP_*-*lacZ*. We selected for mutants with increased resistance to cefoxitin by plating cultures of the wild-type P*_sigP_*-*lacZ* strain (THE2549) on LB-cefoxitin (200 μg/ml) agar. At this concentration of cefoxitin, wild-type B. thuringiensis fails to grow. These strains were tested for P*_sigP_-lacZ* expression in the absence of cefoxitin by streaking on LB–X-Gal. We isolated 8 independent mutants with increased resistance to cefoxitin that have constitutive P*_sigP_-lacZ* expression. We hypothesized that these strains harbored mutations in *rsiP*. We PCR amplified and sequenced the *sigP* and *rsiP* genes from the constitutive mutants. The 8 constitutive mutants contained mutations in different regions of the *rsiP* gene that resulted in C-terminal truncations of RsiP (Fig. S7). We selected four *rsiP* mutants for further study. We found that each mutant strain showed increased P*_sigP_-lacZ* expression even in the absence of β-lactams (Fig. 5). When a wild-type copy of *rsiP* (pSigPRsiP) was introduced to each of these mutants, P*_sigP_*-*lacZ* expression was no longer constitutive but was induced in the presence of cefoxitin (Fig. S8). This indicates that the *rsiP* mutations were responsible for the increased P*_sigP_-lacZ* expression.

10.1128/mSphere.00511-19.7 FIG S7 RsiP mutants result in constitutive σ^P^ activity. Alignment of RsiP mutants that result in nonsense, frameshift, or point mutations. Amino acid residues in red are indicative of the change in amino acid sequence due to mutations. The mutation *rsiP*^1–232^ was isolated 3 independent times while *rsiP*^1–61^ and *rsiP*^1–15^ were isolated twice each. The red box indicates the predicted transmembrane domain. Download FIG S7, TIF file, 1.5 MB. Copyright © 2019 Ho et al. 2019 Ho et al. This content is distributed under the terms of the Creative Commons Attribution 4.0 International license.

10.1128/mSphere.00511-19.8 FIG S8 Complementation of RsiP mutants. B. thuringiensis containing either empty vector (wild type, EBT169; *rsiP*^1–220^, EBT564; *rsiP*^1–80^, EBT565; *rsiP*^1–61^, EBT566; *rsiP*^1–16^, EBT567) or P*_sigP_*-*sigP*-*rsiP* (wild type, EBT168; *rsiP*^1–220^, EBT560; *rsiP*^1–80^, EBT651; *rsiP*^1–61^, EBT562; *rsiP*^1–16^, EBT563) was grown overnight and spotted onto LB–X-Gal (100 μg/ml) plates lacking cefoxitin (A) or containing cefoxitin (5 μg/ml) (B). Download FIG S8, TIF file, 1.8 MB. Copyright © 2019 Ho et al. 2019 Ho et al. This content is distributed under the terms of the Creative Commons Attribution 4.0 International license.

In the σ^V^ and σ^W^ systems, RasP cleaves the anti-σ factors RsiW and RsiV within the transmembrane domain to activate the cognate σ factors (15, 35). The RsiP transmembrane is predicted to be residues 54 to 71 based on TMHMM (44). Two of the four RsiP truncations produce proteins with the transmembrane domain intact, while the remaining RsiP truncations lack the transmembrane domain. Since RasP is known to cleave proteins within the transmembrane domain, we hypothesized that those truncations which still contain a transmembrane domain would require RasP in order to activate σ^P^. To test this, we introduced the *ΔrasP* mutation into each of the *rsiP* mutants. In the absence of RasP, strains containing truncations which have a transmembrane domain (RsiP^1–220^ and RsiP^1–80^) (Fig. 4 and Fig. S7) no longer constitutively activate σ^P^ (Fig. 5). However, the strains with the *rsiP* truncation lacking the transmembrane domain (RsiP^1–16^ and RsiP^1–61^) constitutively activate σ^P^ even in the absence of RasP (RsiP^1–16^ and RsiP^1–61^) (Fig. 4 and Fig. S5). Thus, RasP is required for σ^P^ activation when the transmembrane domain of RsiP is intact, consistent with the role of RasP as a site-2 protease.

### RasP cleaves within the transmembrane domain of RsiP and is not the regulated step in σ^P^ activation.

**FIG 6:**
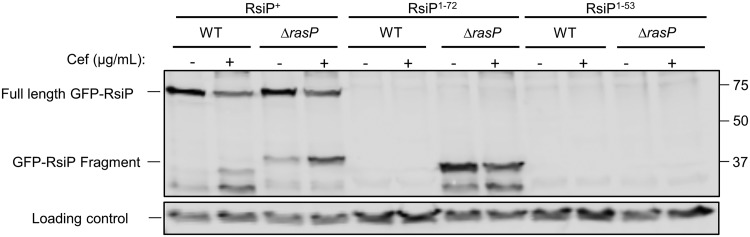
Truncation of RsiP results in constitutive degradation in a RasP-dependent manner. B. thuringiensis containing a tetracycline-inducible copy of *gfp*-*rsiP*, *gfp-rsiP*^1–72^ (*rsiP* without the extracellular domain), or *gfp-rsiP*^1–53^ (*rsiP* without the transmembrane and extracellular domains) was constructed in either the wild type (*rasP*^+^) or a *ΔrasP* mutant strain (GFP-RsiP wild type [*rasP*^+^], EBT360; GFP-RsiP *ΔrasP*, EBT366; GFP-RsiP^1–53^ wild type [*rasP*^+^], EBT518; GFP-RsiP^1–53^ *ΔrasP*, EBT510; GFP-RsiP^1–72^ wild type [*rasP*^+^], EBT516; GFP-RsiP^1–72^ *ΔrasP*, EBT533). Strains were subcultured 1:50 into LB supplemented with ATc (100 ng/ml), grown to mid-log phase, and then incubated for 2 h without (−) or with (+) cefoxitin treatment (5 μg/ml) at 37°C. The immunoblot was probed with anti-GFP antisera. Streptavidin IR680LT was used to detect HD73_4231 (PycA homolog), which served as a loading control (62, 63). The color blot showing both anti-GFP and streptavidin on a single gel is shown in Fig. S9. Numbers at right are molecular masses in kilodaltons.

In the case of σ^W^ and σ^V^, the rate-limiting step in σ factor activation is site-1 cleavage (15, 35). Since the identity of the site-1 protease is not currently known, we sought to determine if RasP cleavage of RsiP is a rate-limiting step in σ^P^ activation. To test this, we constructed truncations of GFP-RsiP that lack the extracellular portion of RsiP. One truncation includes the transmembrane domain (*gfp*-*rsiP*^1–72^), and one truncation lacks the transmembrane domain (*gfp-rsiP*^1–53^). We expressed the truncated GFP-RsiP proteins in wild-type and *ΔrasP* backgrounds and exposed these strains to cefoxitin (5 μg/ml). In wild-type strains, we found that both GFP-RsiP^1–72^ and GFP-RsiP^1–53^ were degraded (Fig. 6 and Fig. S9). However, in the Δ*rasP* mutant GFP-RsiP^1–72^ accumulated, while GFP-RsiP^1–53^ was degraded (Fig. 6 and Fig. S9). These data indicate that GFP-RsiP^1–72^ requires RasP for degradation while GFP-RsiP^1–53^ does not. One possible interpretation is that GFP-RsiP^1–72^ is not produced or localized properly to the membrane. Thus, we confirmed that GFP-RsiP^1–72^ localizes to the membrane by fluorescence microscopy (Fig. S2). This suggests that the RasP cleavage site of RsiP occurs within the transmembrane domain between amino acids 53 and 72. The presence or absence of cefoxitin had no effect on the degradation (Fig. 6 and Fig. S9). Since GFP-RsiP^1–72^ is constitutively degraded, we conclude that GFP-RsiP^1–72^ mimics the site-1 cleavage product and that RasP activity is not induced by cefoxitin. This suggests that RasP cleavage of RsiP is not the regulated step in σ^P^ activation and that site-1 cleavage is the step that is controlled by the presence of β-lactams.

10.1128/mSphere.00511-19.9 FIG S9 Truncation of RsiP results in constitutive degradation in a RasP-dependent manner. B. thuringiensis containing a tetracycline-inducible copy of *gfp*-*rsiP*, *gfp-rsiP*^1–72^ (*rsiP* without the extracellular domain) or *gfp-rsiP*^1–53^ (*rsiP* without the transmembrane and extracellular domains), was constructed in either wild type or a *ΔrasP* mutant strain (GFP-RsiP wild type [*rasP*^+^], EBT360; GFP-RsiP *ΔrasP*, EBT366; GFP-RsiP^1–53^ wild type [*rasP*^+^], EBT518; GFP-RsiP^1–53^ *ΔrasP*, EBT510; GFP-RsiP^1–72^ wild type [*rasP*^+^], EBT516; GFP-RsiP^1–72^ *ΔrasP*, EBT533). Strains were subcultured 1:50 into LB supplemented with ATc (100 ng/ml) and at mid-log phase were incubated for 2 h without (−) or with (+) cefoxitin treatment (5 μg/ml) at 37°C. The immunoblot was probed with antisera against GFP (anti-GFP) followed by goat anti-rabbit IgG IR800CW (green). Streptavidin IR680LT (red) was used to detect HD73_4231 (PycA homolog), which served as a loading control (62, 63). Download FIG S9, TIF file, 1.7 MB. Copyright © 2019 Ho et al. 2019 Ho et al. This content is distributed under the terms of the Creative Commons Attribution 4.0 International license.

## DISCUSSION

Many ECF σ factors are induced in response to extracytoplasmic stressors and initiate transcription of a subset of genes to modulate the cell’s response to these stresses. ECF σ factors can respond to signals such as misfolded periplasmic protein, antimicrobial peptides, or lysozyme. The ECF σ factors encoded in highly related organisms can vary widely. For example, B. subtilis encodes 7 ECF σ factors, while B. thuringiensis encodes 15 predicted ECF σ factors. The only ECF σ factor that these organisms share is σ^M^ (45). Thus, there is a variability in how bacteria utilize ECF σ factors to respond to stress. Ross et al. demonstrated that the novel ECF σ factor σ^P^ is induced in the presence of ampicillin and initiates transcription of β-lactamases (5). Here, we demonstrated that σ^P^ responds specifically to a subset of β-lactams, while other β-lactams and cell wall-targeting antibiotics fail to induce σ^P^ activation. We also showed that σ^P^ confers various degrees of resistance to these β-lactam antibiotics. We found that σ^P^ was not required for resistance to other cell wall antibiotics, including vancomycin, nisin, and bacitracin, suggesting specificity in resistance to β-lactams and not a general cell envelope stress response.

For ECF σ factors to be activated, their cognate anti-σ factors must be inactivated. This can be accomplished via various mechanisms, including a conformational change of the anti-σ factor; partner switching, where an anti-anti-σ factor frees the σ factor from the anti-σ factor; or proteolytic destruction of the anti-σ factor (9, 11). The anti-σ factors RseA in E. coli and RsiW and RsiV in B. subtilis are degraded sequentially by regulated intramembrane proteolysis. Each of these anti-σ factors requires a different family of proteases to cleave the anti-σ factor at site-1 (14, 22, 30, 46, 47), while site-2 cleavage is carried out by the conserved site-2 protease (14, 15, 35). We hypothesize that σ^P^ is activated in a similar manner. Our data indicate that σ^P^ is released from RsiP by proteolytic degradation when β-lactams are present. We found that RasP is required for activation of σ^P^. We also observe that an RsiP degradation product approximately the size of our predicted RasP substrate accumulates in a *ΔrasP* mutant. This indicates that RasP is required for degradation of RsiP. Our data also suggest, similarly to other anti-σ factors, that site-2 cleavage of RsiP is not the rate-limiting step, since the C-terminal RsiP truncations are constitutively degraded and lead to constitutive σ^P^ activation in the absence of β-lactams. Thus, we hypothesize that RasP is required for site-2 cleavage of RsiP and that an as-yet-unidentified protease is required to initiate degradation of RsiP by cleaving RsiP at site-1. We hypothesize that, like other ECF σ factors activated by regulated intramembrane proteolysis, site-1 cleavage of RsiP is likely the rate-limiting step in σ^P^ activation.

Our data suggest that a subset of β-lactams induce σ^P^ activation. We found that, in addition to ampicillin, σ^P^ is activated by cefoxitin, cefmetazole, cephalothin, cephalexin, and methicillin but not by piperacillin, cefoperazone, cefsulodin, or antibiotics that target other steps in peptidoglycan biosynthesis. This raises the question of what the signal is for σ^P^ activation. The β-lactams could be sensed directly or indirectly. For example, RsiV directly senses lysozyme and degradation of RsiV is rapid (31). In contrast, activation of σ^E^ is indirect and due to buildup of products that occur when the outer membrane is damaged (31, 48). Our data suggest that RsiP degradation is a relatively slow process. One possible interpretation of this is that β-lactam-induced peptidoglycan (PG) damage must accumulate to induce RsiP degradation. We hypothesize that the β-lactams that we tested have different affinities for penicillin-binding proteins (PBPs) and that this affinity may explain why some β-lactams induce σ^P^ while others do not. In other organisms, including Streptococcus pneumoniae, B. subtilis, and E. coli, β-lactams can differentially target PBPs (49–51). This raises the possibility that activation of σ^P^ could be the result of inhibition of specific PBPs. Unfortunately, at this time we do not know which PBPs are targeted by the different β-lactams in B. thuringiensis. Thus, the precise mechanism and signal responsible for σ^P^ activation remain to be clearly defined.

## MATERIALS AND METHODS

### Media and growth conditions.

All B. thuringiensis strains are isogenic derivatives of AW43, a derivative of Bacillus thuringiensis subsp. *kurstaki* strain HD73 (52). All strains and genotypes can be found in Table 3. All B. thuringiensis strains were grown in or on LB medium at 30°C unless otherwise specified. Cultures of B. thuringiensis were grown with agitation in a roller drum. Strains containing episomal plasmids were grown in LB containing chloramphenicol (Cam; 10 μg/ml) or erythromycin (Erm; 10 μg/ml). E. coli strains were grown at 37°C using LB-ampicillin (Amp; 100 μg/ml) or LB-Cam (10 μg/ml) medium. To screen for threonine auxotrophy, B. thuringiensis strains were patched on minimal medium plates without or with threonine (50 μg/ml) (53, 54). The β-galactosidase chromogenic indicator 5-bromo-4-chloro-3-indolyl-β-d-galactopyranoside (X-Gal) was used at a concentration of 100 μg/ml. Anhydrotetracycline (ATc; Sigma) was used at a concentration of 100 ng/ml.

**TABLE 3:** Strains

Species and strain	Description	Reference or source
B. thuringiensis		
AW43	B. thuringiensis subsp. *kurstaki* HD73 cured of both pAW63 and pHT73, Nal^r^	52
THE2549	AW43 *thrC*::P*_sigP_-lacZ*	This study
EBT140	AW43 *thrC*::P*_sigP_-lacZ* Δ*rasP*	This study
EBT232	AW43 *thrC*::P*_sigP_*-*lacZ* Δ*sigP-rsiP*	This study
EBT215	AW43 *thrC*::P*_sigP_*-*lacZ* Δ*bla1*	This study
EBT360	AW43 *thrC*::P*_sigP_*-*lacZ*/pAH9 P*_tet_*-*gfp*-*rsiP*	This study
EBT366	AW43 *thrC*::P*_sigP_*-*lacZ* Δ*rasP*/pAH9 P*_tet_*-*gfp*-*rsiP*	This study
EBT510	AW43 *thrC*::P*_sigP_*-*lacZ* Δ*rasP*/pAH9 P*_tet_*-*gfp*-*rsiP*^1–53^	This study
EBT516	AW43 *thrC*::P*_sigP_*-*lacZ*/pAH9 P*_tet_*-*gfp*-*rsiP*^1–72^	This study
EBT518	AW43 *thrC*::P*_sigP_*-*lacZ*/pAH9 P*_tet_*-*gfp*-*rsiP*^1–53^	This study
EBT533	AW43 *thrC*::P*_sigP_*-*lacZ* Δ*rasP*/pAH9 P*_tet_*-*gfp*-*rsiP*^1–72^	This study
EBT175	AW43 *thrC*::P*_sigP_*-*lacZ* Δ*rasP*/pAH9	This study
EBT176	AW43 *thrC*::P*_sigP_*-*lacZ* Δ*rasP*/pAH9 *rasP*	This study
EBT238	AW43 *thrC*::P*_sigP_*-*lacZ* Δ*sigP-rsiP*/pAH9 P*_sigP_*-*sigP*-*rsiP*	This study
EBT251	AW43 *thrC*::P*_sigP_*-*lacZ* Δ*sigP-rsiP*/pAH9	This study
THE2642	AW43 *thrC*::P*_sigP_*-*lacZ rsiP*^1–16^	This study
THE2637	AW43 *thrC*::P*_sigP_*-*lacZ rsiP*^1–61^	This study
THE2628	AW43 *thrC*::P*_sigP_*-*lacZ rsiP*^1–80^	This study
THE2602	AW43 *thrC*::P*_sigP_*-*lacZ rsiP*^1–220^	This study
THE2605	AW43 *thrC*::P*_sigP_*-*lacZ* Δ*rasP rsiP*^1–16^	This study
EBT133	AW43 *thrC*::P*_sigP_*-*lacZ* Δ*rasP rsiP*^1–61^	This study
EBT148	AW43 *thrC*::P*_sigP_*-*lacZ* Δ*rasP rsiP*^1–80^	This study
EBT116	AW43 *thrC*::P*_sigP_*-*lacZ* Δ*rasP rsiP*^1–220^	This study
EBT567	AW43 *thrC*::P*_sigP_*-*lacZ rsiP*^1–16^/pAH9 P*_sigP_*-*sigP*-*rsiP*	This study
EBT566	AW43 *thrC*::P*_sigP_*-*lacZ rsiP*^1–61^/pAH9 P*_sigP_*-*sigP*-*rsiP*	This study
EBT565	AW43 *thrC*::P*_sigP_*-*lacZ rsiP*^1–80^/pAH9 P*_sigP_*-*sigP*-*rsiP*	This study
EBT564	AW43 *thrC*::P*_sigP_*-*lacZ rsiP*^1–220^/pAH9 P*_sigP_*-*sigP*-*rsiP*	This study
EBT168	AW43 *thrC*::P*_sigP_*-*lacZ*/pAH9 P*_sigP_*-*sigP*-*rsiP*	This study
EBT169	AW43 *thrC*::P*_sigP_*-*lacZ* pAH9	This study
EBT563	AW43 *thrC*::P*_sigP_*-*lacZ rsiP*^1–16^/pAH9	This study
EBT562	AW43 *thrC*::P*_sigP_*-*lacZ rsiP*^1–61^/pAH9	This study
EBT561	AW43 *thrC*::P*_sigP_*-*lacZ rsiP*^1–80^/pAH9	This study
EBT560	AW43 *thrC*::P*_sigP_*-*lacZ rsiP*^1–220^/pAH9	This study
UM20	AW43/pAH13	This study
EBT587	AW43 *thrC*::P*_sigP_*-*lacZ rsiP*^1–80^/pAH9 P*_tet_*-*gfp*-*rsiP*	This study

E. coli		
OmniMax 2-T1R	F′ {*proAB*^+^ *lacI*^q^ *lacZ*ΔM15 Tn*10*(Tet^r^) Δ(*ccdAB*)} *mcrA* Δ(*mrr*-*hsdRMS*-*mcrBC*) ϕ80(*lacZ*)ΔM15 Δ(*lacZYA*-*argF*)*U169* *endA1 recA1 supE44 thi-1 gyrA96 relA1 tonA panD*	Invitrogen
INV110	*endA1 rpsL thr leu thi lacY galK galT ara tomA tsx dam* *dcm supE44* Δ(*lac*-*proAB*) [F′ *traD36 proAB lacI*^q^*Z*ΔM15]	Invitrogen

### Strain and plasmid construction.

All plasmids are listed in Table 4, which includes information relevant to plasmid assembly. Plasmids were constructed by isothermal assembly (55). Regions of plasmids constructed using PCR were verified by DNA sequencing. The oligonucleotide primers used in this work were synthesized by Integrated DNA Technologies (Coralville, IA) and are listed in Table S1 in the supplemental material. All plasmids were propagated using OmniMax 2-T1R as the cloning host and passaged through the nonmethylating E. coli strain INV110 before being transformed into a B. thuringiensis recipient strain.

**TABLE 4:** Plasmids

Plasmid	Relevant feature(s)	Parent vector	Digestion enzymes	Insert primers	Reference
pMAD	ori-pE194ts				56
pAH9	ori-pE194 P*_sarA_-mcherry*				57
pAH13	P*_tet_-gfp*				57
pRAN332	P*_tet_-gfp*				64
pEBT4	ori-pE194ts, Δ*blaP*	pMAD	BgIII, EcoRI	3832 and 3833, 3834 and 3835	This study
pEBT5	ori-pE194ts, Δ*rasP*	pMAD	BgIII, EcoRI	3632 and 3633, 3634 and 3635	This study
pEBT6	ori-pE194ts, Δ*sigP-rsiP*	pMAD	BgIII, EcoRI	3776 and 3777, 3778 and 3779	This study
pEBT13	P*_tet_-gfp-rsiP*	pAH9	HindIII, EcoRI	3838 and 3839	This study
pCE630	P*_tet_-gfp-rsiP*^1–72^	pAH9	HindIII, EcoRI	3838 and 4258	This study
pCE632	P*_tet_-gfp-rsiP*^1–53^	pAH9	HindIII, EcoRI	3838 and 4259	This study
pTHE960	P*_sigP_-sigP^+^*-*rsiP^+^*	pAH9	HindIII, EcoRI	3774 and 3775	This study
pIA02	P*_sarA_-rasP^+^*	pAH9	EcoRI, KpnI	3744 and 3745	This study
pTHE946	pE194ts	pMAD	BamHI, StuI		This study
pTHE948	pE194ts ‘*thrC thrB*’	pTHE946	ScaI, SalI	2917 and 2918, 2919 and 2920	This study
pTHE950	pE194ts ‘*thrC lacZ thrB*’	pTHE948	XhoI, SbfI	2922 and 2923	This study
pTHE949	pE194ts ‘*thrC* P*_sigP_-lacZ thrB*’	pTHE950	XhoI, SalI	2929 and 2930	This study

10.1128/mSphere.00511-19.10 TABLE S1 Oligonucleotides. Download Table S1, PDF file, 0.06 MB. Copyright © 2019 Ho et al. 2019 Ho et al. This content is distributed under the terms of the Creative Commons Attribution 4.0 International license.

To construct deletion mutants, we cloned DNA 1 kb upstream and 1 kb downstream of the site of desired deletion using primers listed in Table S1 onto the temperature-sensitive pMAD plasmid (erythromycin resistant) between the BglII and EcoRI sites (56).

Complementation constructs were constructed in pAH9, which is an E. coli–Gram-positive bacterial shuttle vector with a pE194 origin of replication (57). Chromosomal DNA including the promoter sequence was cloned for P*_sigP_-sigP^+^*-*rsiP^+^* and cloned into pAH9 digested with EcoRI and HindIII, while *rasP* was cloned downstream of the P*_sarA_* promoter from Staphylococcus aureus by digesting with EcoRI and KpnI. In B. thuringiensis, P*_sarA_* has moderate constitutive expression.

To generate strains containing the *sigP* promoter fused to the *lacZ* reporter integrated into the chromosome, we constructed a number of intermediate vectors. To switch the antibiotic resistance of the temperature-sensitive pMAD vector, we constructed pTHE946, which contains the E. coli origin (ColE1 ori) of replication, an Erm resistance gene (for selection in Gram-positive bacteria), an Amp resistance gene (for selection in E. coli strains), and the temperature-sensitive origin (pE194 ori) from pMAD (7.3-kb StuI and BamHI fragment) as well as the conjugation origin of transfer and the Cam resistance gene from pRPF185 (SmaI and BamHI fragment). The *thrC* (primers 2917 and 2918) and *thrB* (primers 2919 and 2920) genes were cloned into the ScaI- and SalI-digested pTHE946 plasmid (lacking Erm^r^ and Amp^r^ genes) to generate a vector (pTHE948) which can integrate into the *thrC* operon. A promoterless *lacZ* fragment (primers 2922 and 2923) was added between the *thrC* and *thrB* genes of pTHE948 (XhoI and SbfI) to generate pTHE950. This plasmid (XhoI and NotI digested) was used to clone the *sigP* promoter (primers TE2929 and 2930) to generate the P*_sigP_*-*lacZ* promoter fusion (pTHE949).

### B. thuringiensis DNA transformation.

Plasmids were introduced into B. thuringiensis by electroporation (58, 59). Briefly, recipient cells were grown to late log phase at 37°C. For each transformation, cells (1.5 ml) were pelleted by centrifugation (9,000 × *g*) and washed twice in room-temperature sterile water. After careful removal of all residual water, 100 μl of sterile 40% polyethylene glycol (PEG) 6000 (Sigma) was used to gently resuspend cells. Approximately 2 to 10 μl of unmethylated DNA (>50 ng/μl) was added to cells and transferred to an 0.4-cm-gap electroporation cuvette (Bio-Rad). Cells were exposed to 2.5 kV for 4 to 6 ms. LB was immediately added, and cells were incubated at 30°C for 1 to 2 h prior to plating on selective media.

### Construction of deletions or promoter-*lacZ* fusions in B. thuringiensis.

To generate unmarked mutants and *thrC*::P*_sigP_-lacZ* strains, we used plasmid vectors containing the temperature-sensitive origin of replication (pE194 ori) from the pMAD plasmid (56). At permissive temperatures (30°C), pMAD replicates episomally as a plasmid. At nonpermissive temperatures (42°C), pMAD must integrate into the chromosome via homologous recombination; otherwise, the plasmid will be lost to segregation and the strain will become sensitive to erythromycin. Plasmids were transformed into a B. thuringiensis recipient strain and selected for on LB-Erm agar at 30°C. To select for the integration of the deletion plasmid into the recipient strain genome, plasmid-containing bacteria were grown at 42°C on LB-Erm plates. The plasmid-integrated strain was then struck on LB agar at 30°C twice. Individual colonies were patched on LB and LB-Erm agar to identify the Erm-sensitive bacteria which had lost the deletion plasmid by segregation. To verify each deletion, genomic DNA was isolated from each strain candidate and PCR was used to verify the deletion. Integration of the P*_sigP_-lacZ* fusion into the *thrC* operon results in threonine auxotrophy and can be identified by lack of growth on minimal medium plates without threonine.

### Zones of inhibition and zones of induction.

To determine the zones of inhibition and induction by various antibiotics, we first washed mid-logarithmically grown cells in fresh LB. Washed cells were diluted 1:100 in molten LB agar containing X-Gal (100 μg/ml) and poured into empty 100-mm petri dishes. Sterile cellulose disks (8 mm) were saturated with different antibiotics and allowed to dry for longer than 10 min. After each antibiotic disk was placed on the solidified agar, plates were incubated at 30°C overnight before observation.

### β-Galactosidase assays.

To quantify expression from the *sigP* promoter, we measured the β-galactosidase activity of cells containing a P*_sigP_*-*lacZ* promoter fusion. Overnight cultures were diluted 1:50 in fresh LB medium and incubated for 3 h at 30°C. One milliliter of each subculture was pelleted (9,000 × *g*), washed (in LB broth), and resuspended in 1 ml LB broth lacking or including specified antibiotics. After 1 h of incubation at 37°C, 1 ml of each sample was pelleted and resuspended in 200 μl of Z buffer. Cells were permeabilized by mixing with 16 μl chloroform and 16 μl 2% Sarkosyl (26, 60). Permeabilized cells (100 μl) were mixed with 10 mg/ml *ortho*-nitrophenyl-β-galactoside (ONPG; Research Products International; 50 μl), and optical density at 405 nm (OD_405_) was measured over time using an Infinite M200 Pro plate reader (Tecan). β-Galactosidase activity units (μmol of ONP formed min^−1^) × 10^3^/(OD_600_ × ml of cell suspension) were calculated as previously described (61). Experiments were performed in triplicate with the mean and standard deviation being shown.

### MIC assay.

To determine the MICs of various antibiotics, we diluted overnight cultures of bacteria (washed in LB) 1:1,000 in medium containing 2-fold dilutions of each antibiotic. All MIC experiments were performed in round-bottom 96-well plates. Each experiment was performed in triplicate, and the plates were allowed to incubate for 24 h at 37°C before observation of growth or no growth.

### Immunoblot analysis.

Samples were electrophoresed on a 15% SDS-polyacrylamide gel, and proteins were then blotted onto a nitrocellulose membrane (GE Healthcare, Amersham). Nitrocellulose was blocked with 5% bovine serum albumin (BSA), and proteins were detected with either 1:10,000 anti-GFP or 1:5,000 anti-RsiP^76–275^ antiserum. Streptavidin IR680LT (1:10,000) was used to detect two biotin-containing proteins, PycA (HD73_4231) and AccB (HD73_4487), which served as loading controls (62, 63). To detect primary antibodies, the blots were incubated with 1:10,000 goat anti-rabbit IR800CW (Li-Cor) and imaged on an Odyssey CLx scanner (Li-Cor) or an Azure Sapphire imager (Azure Biosystems). All immunoblot assays were performed a minimum of three times with a representative example being shown.
