# Supplementary figures and images for: Activation of the Extracytoplasmic Function σ Factor σP by β-Lactams in Bacillus thuringiensis Requires the Site-2 Protease RasP
Source: mSphere. 2019 Aug 7;4(4):e00511-19. doi: 10.1128/mSphere.00511-19 (PMC6686233; doi:10.1128/mSphere.00511-19)

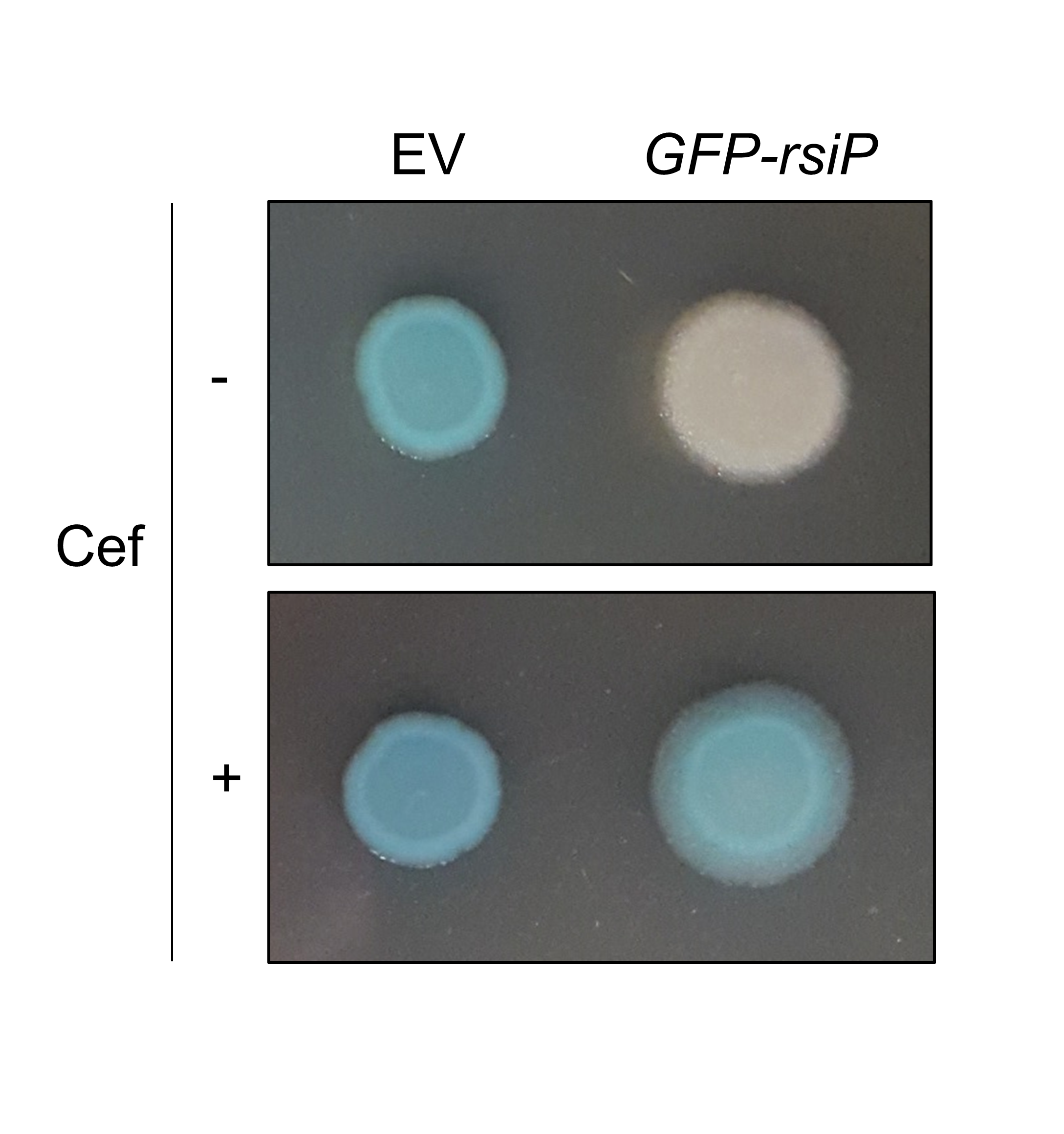

Supplement: FIG S1 [file mSphere.00511-19-sf001.tif]

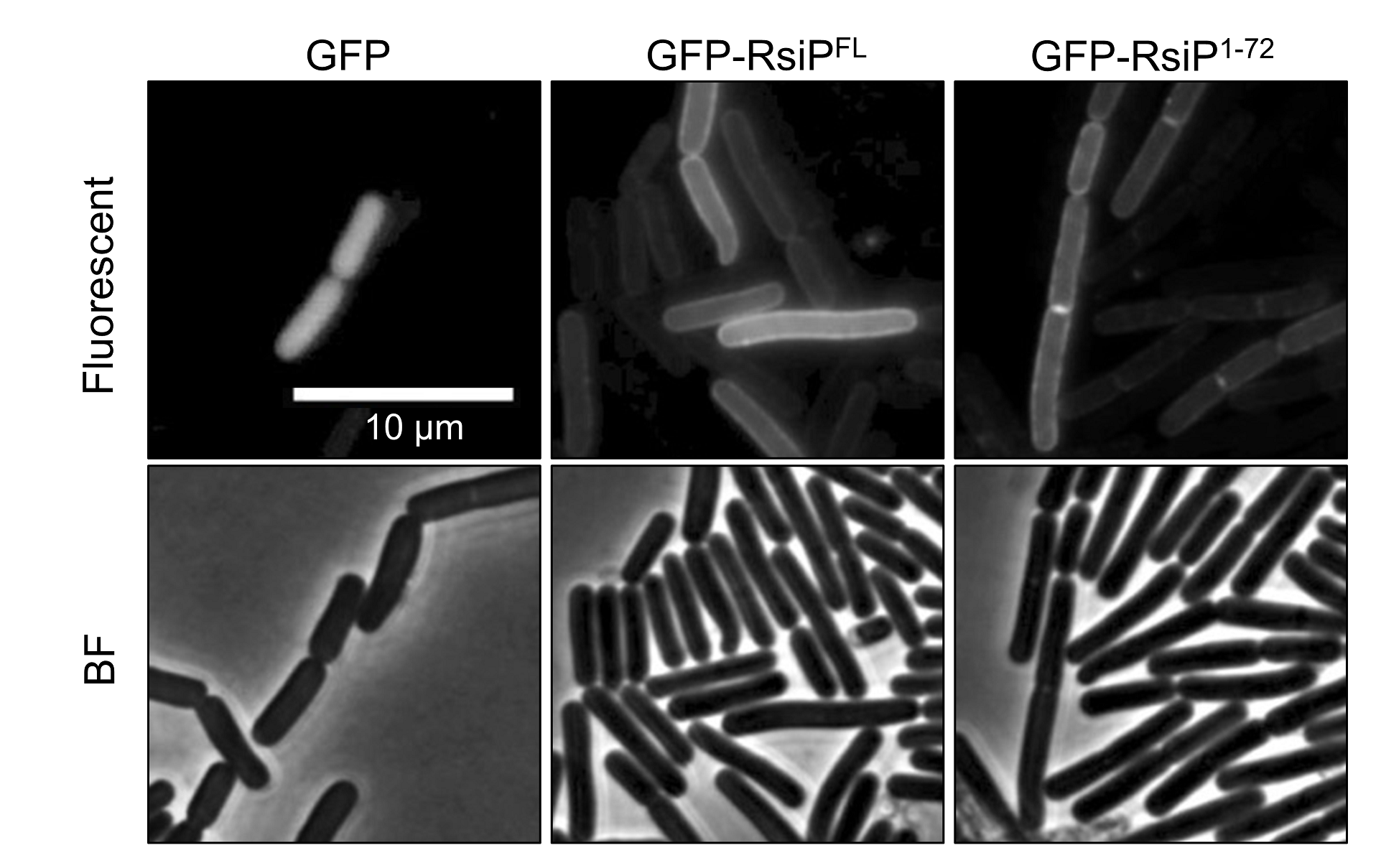

Supplement: FIG S2 [file mSphere.00511-19-sf002.tif]

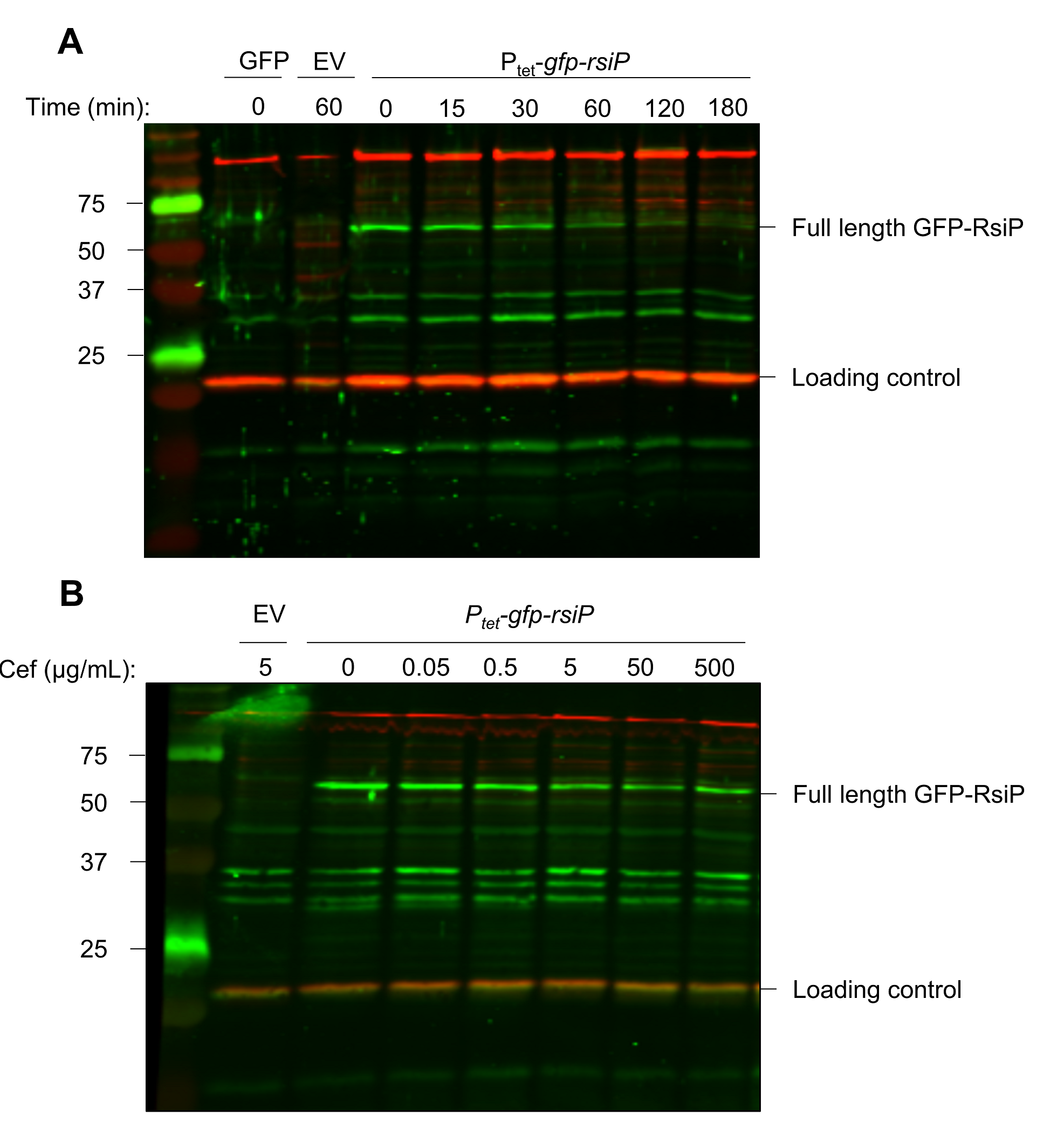

Supplement: FIG S3 [file mSphere.00511-19-sf003.tif]

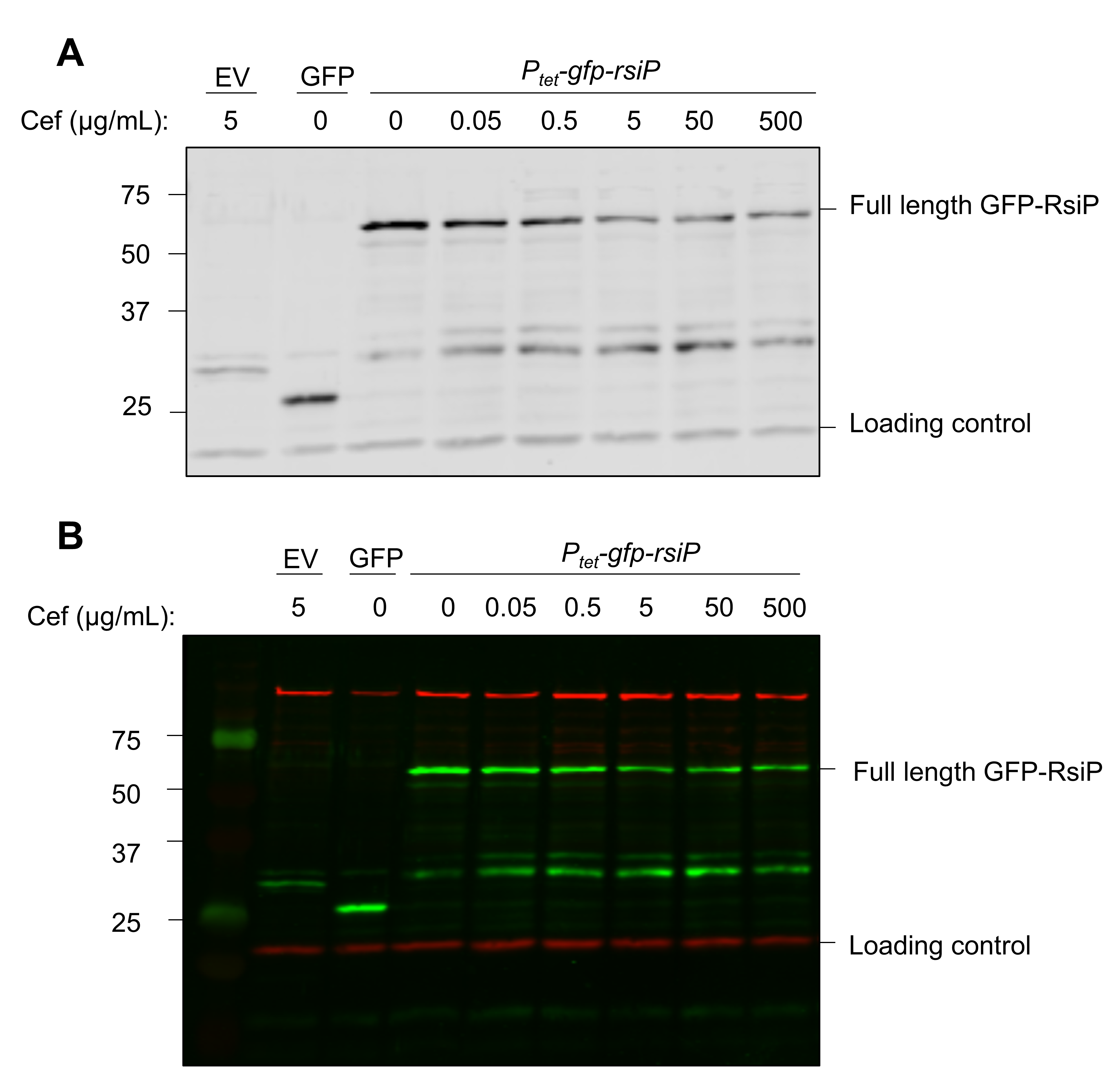

Supplement: FIG S4 [file mSphere.00511-19-sf004.tif]

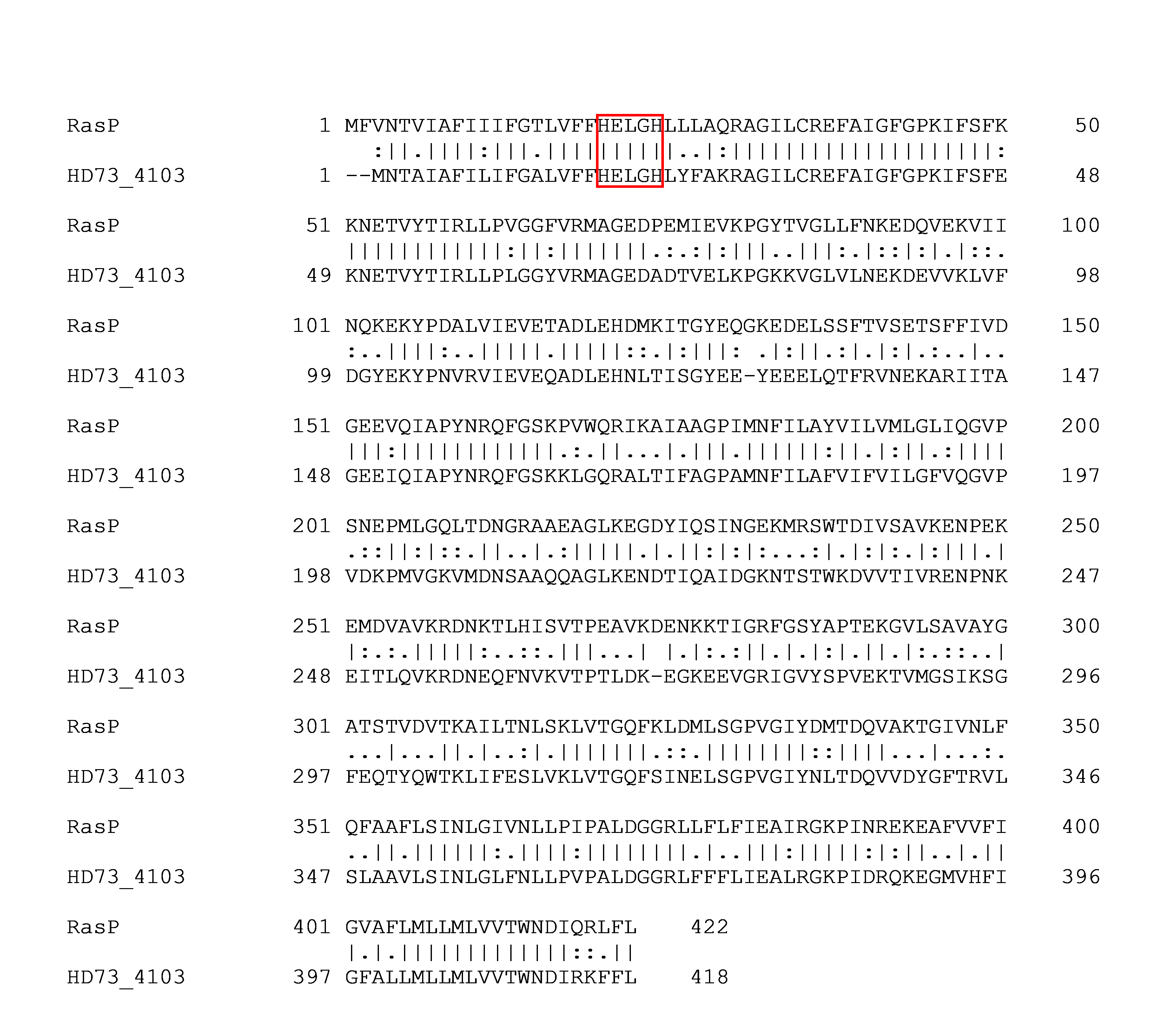

Supplement: FIG S5 [file mSphere.00511-19-sf005.tif]

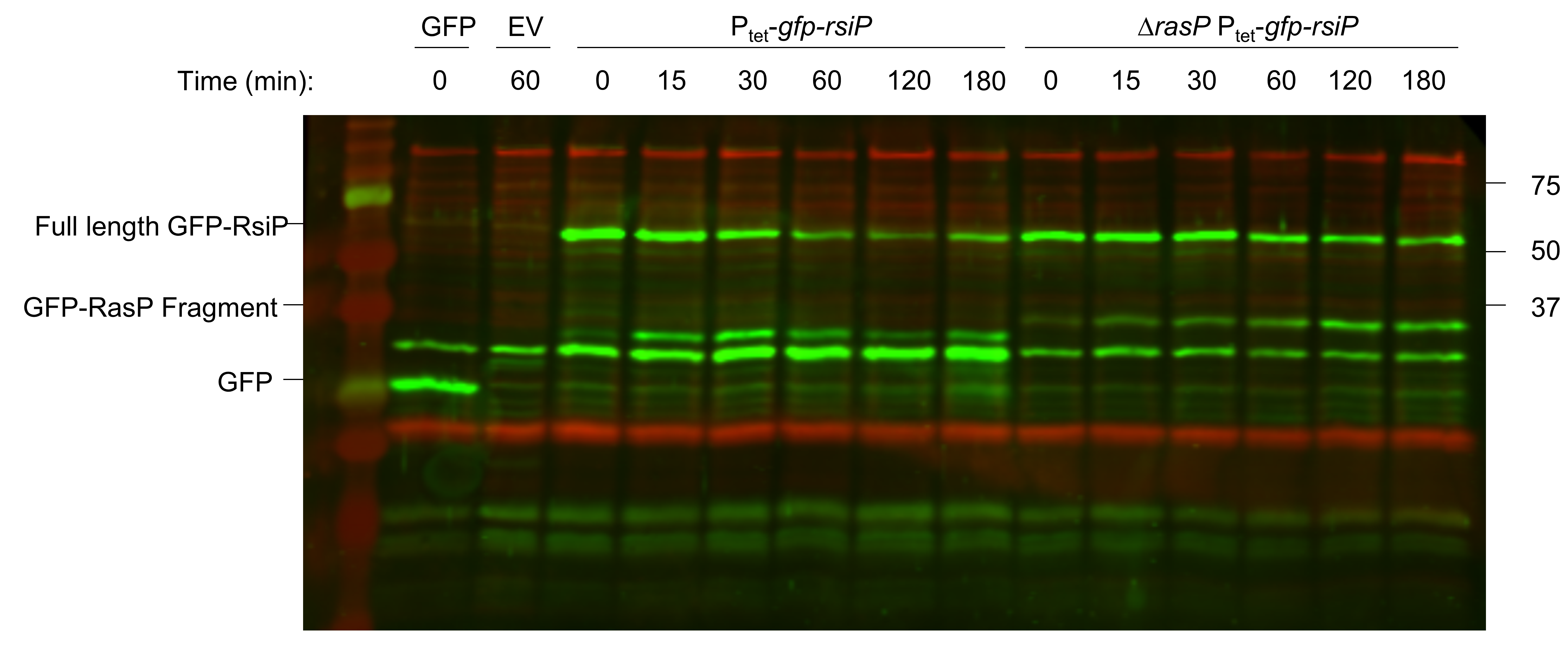

Supplement: FIG S6 [file mSphere.00511-19-sf006.tif]

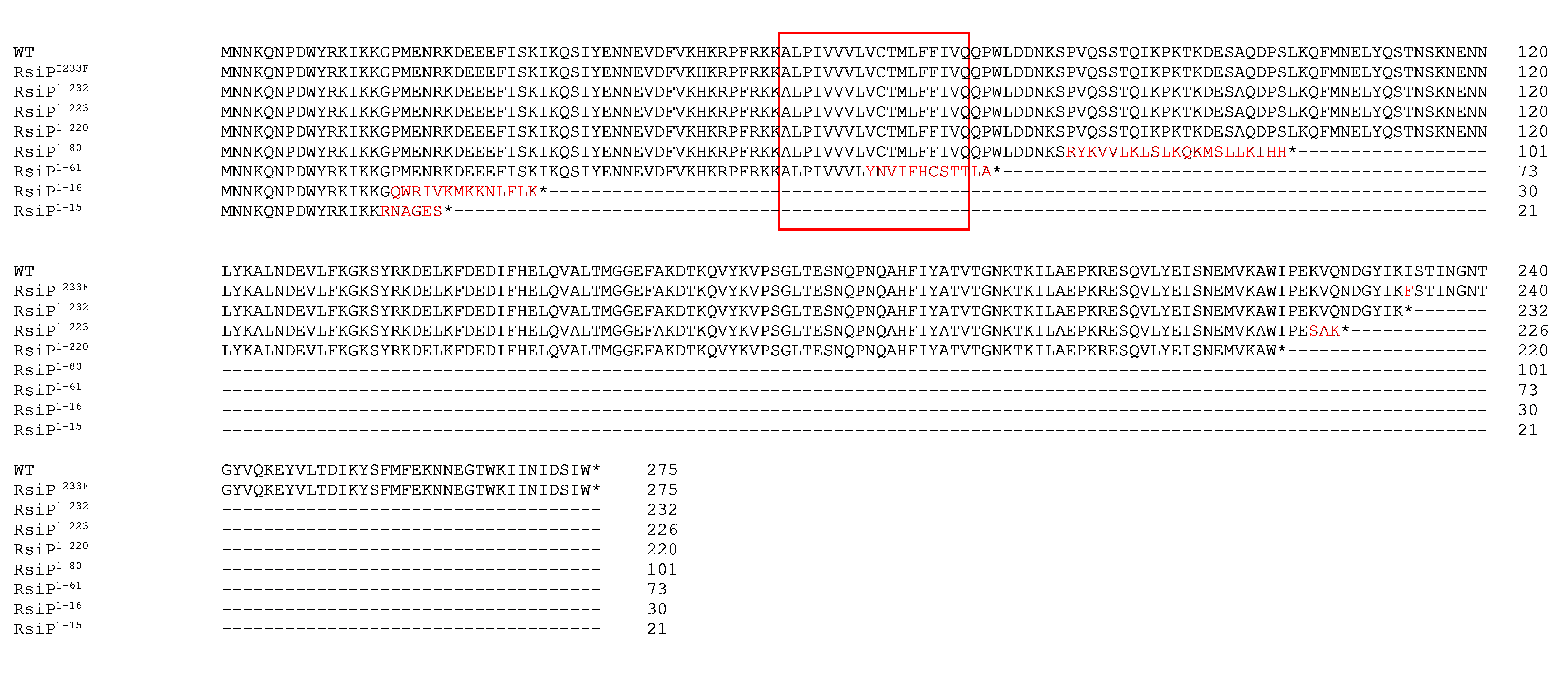

Supplement: FIG S7 [file mSphere.00511-19-sf007.tif]

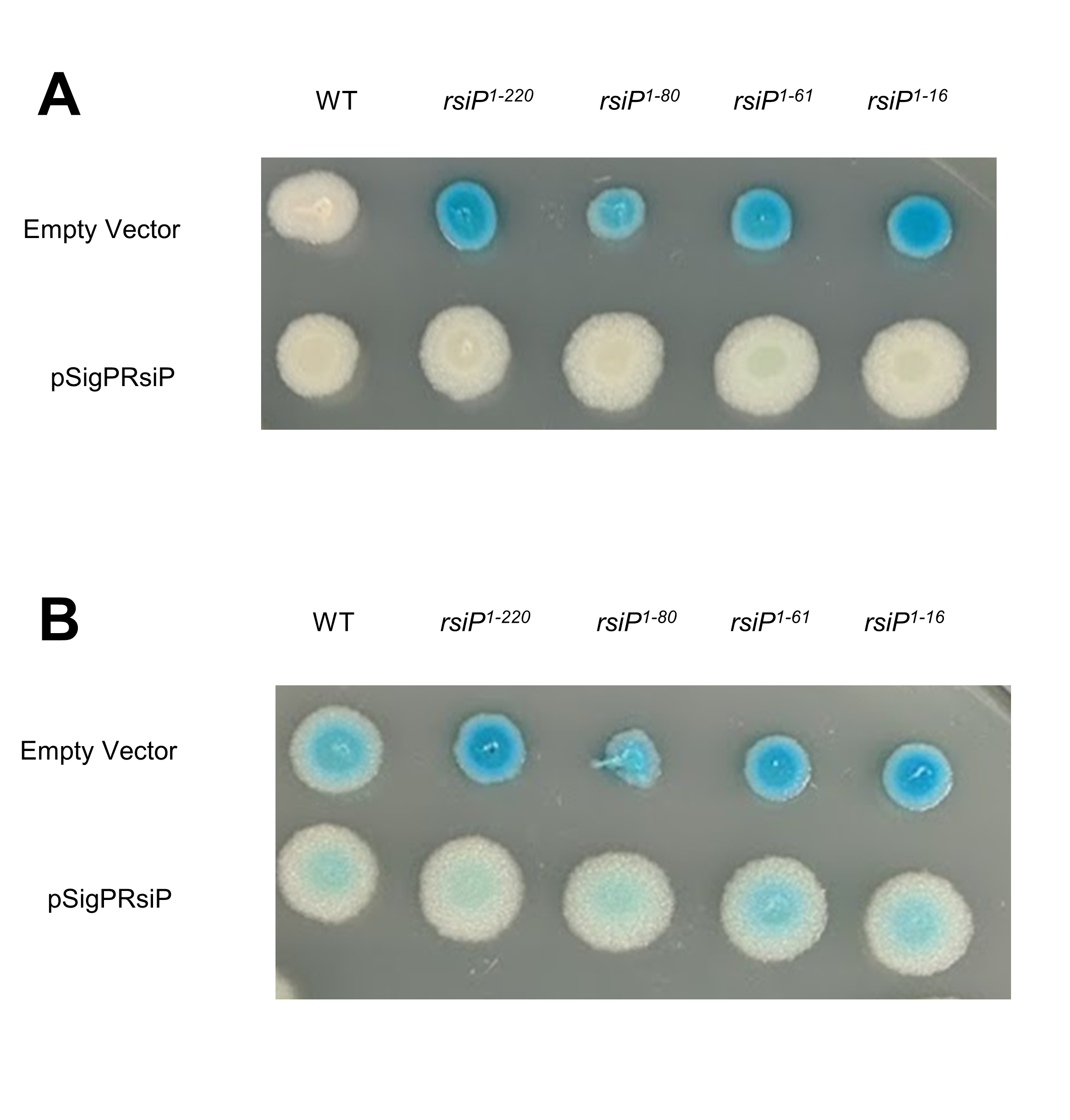

Supplement: FIG S8 [file mSphere.00511-19-sf008.tif]

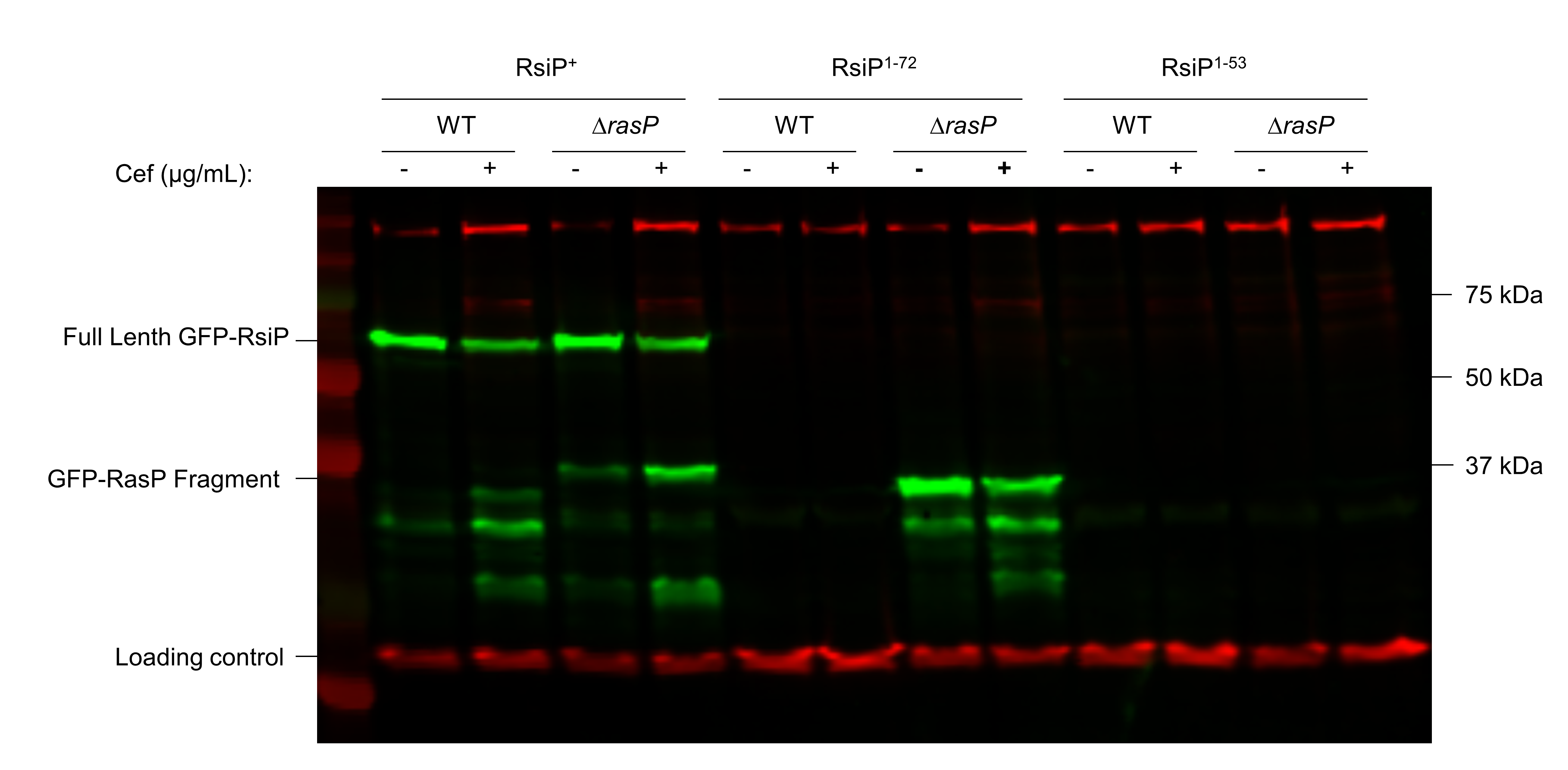

Supplement: FIG S9 [file mSphere.00511-19-sf009.tif]
