# Supplementary material for: Activation of the Extracytoplasmic Function σ Factor σP by β-Lactams in Bacillus thuringiensis Requires the Site-2 Protease RasP
Source: mSphere. 2019 Aug 7;4(4):e00511-19. doi: 10.1128/mSphere.00511-19 (PMC6686233; doi:10.1128/mSphere.00511-19)
Supplement: TABLE S1 [file mSphere.00511-19-st001.pdf]

Table S1 Oligonucleotides

Oligo	Sequence
3632	acacattaactagacagatcggcacttgtttacaacgtgt
3633	ttataagtttcactttatttaaccacctttgctaaatttgttcc
3634	caaatttagcaaaggtggttaaataaagtgaaacttataatgaaatagagc
3635	ctgcagaagcttctagaatttgtacgagatacaccgatac
3776	acacattaactagacagatcaatgaagtactctgataatatttattttgc
3777	ttaaagtgaaccttcctcttttccacacctcaaacttatttg
3778	aataagtttgaggtgtggaaaagaggaaggttcactttaatga
3779	ctgcagaagcttctagaattctatggccaacacggcagtt
3838	acgacggccagtgccaagctctagcataaaaataagaagcctgc
3839	tatgaccatgattacgaattccttcctcttttaccatatagaatc
4258	tatgaccatgattacgaattcttattgttgaacaatgaaaaataacattgtac
4259	tatgaccatgattacgaattcttattttttacgaaatggtcttttatgttttac
2917	gctagaacatctggtgcatt
2918	accctgcaggctcgagaaaaaaaaccgg
2919	ttttctcgagcctgcagggtggttaaagaaaaagctga
2920	tgttcatatttatcagagctcgtaggattactttttaattcactttg
2922	gctttttctttaaccaccctgcaggttatttttgacaccagaccaac
2923	gcaatgcccggttttttttctcgagcggccgcggatccccagcttgttgataca
2929	tcaacaagctggggatccgcggccgcacctcaaacttatttgaataaac
2930	gcaatgcccggttttttttctcgagtaatacttaattatatggtgaattttttg
3774	acgacggccagtgccaagctaattatatggtgaattttttgactaaattt
3907	ggattttgtttattgttcatttccacacctcaaacttatttg
3908	aataagtttgaggtgtggaaatgaacaataaacaaaatcctgac
3909	gctatgaccatgattacgaattttaccatatagaatctatatttattattttcca
